# Advanced Role of Neutrophils in Common Respiratory Diseases

**DOI:** 10.1155/2017/6710278

**Published:** 2017-05-15

**Authors:** Jinping Liu, Zhiqiang Pang, Guoqiang Wang, Xuewa Guan, Keyong Fang, Ziyan Wang, Fang Wang

**Affiliations:** ^1^Institute of Frontier Medical Science, Jilin University, Changchun 130021, China; ^2^Department of Pathogen Biology, College of Basic Medical Sciences, Jilin University, Changchun 130021, China; ^3^Department of Pharmacology, College of Basic Medical Sciences, Jilin University, Changchun 130021, China

## Abstract

Respiratory diseases, always being a threat towards the health of people all over the world, are most tightly associated with immune system. Neutrophils serve as an important component of immune defense barrier linking innate and adaptive immunity. They participate in the clearance of exogenous pathogens and endogenous cell debris and play an essential role in the pathogenesis of many respiratory diseases. However, the pathological mechanism of neutrophils remains complex and obscure. The traditional roles of neutrophils in severe asthma, chronic obstructive pulmonary diseases (COPD), pneumonia, lung cancer, pulmonary fibrosis, bronchitis, and bronchiolitis had already been reviewed. With the development of scientific research, the involvement of neutrophils in respiratory diseases is being brought to light with emerging data on neutrophil subsets, trafficking, and cell death mechanism (e.g., NETosis, apoptosis) in diseases. We reviewed all these recent studies here to provide you with the latest advances about the role of neutrophils in respiratory diseases.

## 1. Introduction

With the changing of global environment, especially the increased air pollution worldwide, respiratory diseases are becoming a main killer to human health. According to recent researches, asthma is ranked as the 14th most important chronic disease, affecting 334 million individuals of all ages worldwide [1]. Lung is the leading cancer site in males, comprising 17% of the total new cancer cases and 23% of the total cancer deaths [2]. As for COPD, affecting 64 million people all over the world, it would be the third most common cause of death by 2030 [3, 4]. Community-acquired pneumonia is a common cause of sepsis, leading to 10 million deaths annually [5]. While epidemiology data of idiopathic pulmonary fibrosis (IPF) worldwide cannot be obtained, IPF incidence is still increasing and carries a high risk of respiratory failure and death [6]. Respiratory diseases not only increase the economic burden of global health care but also cause a terrible effect on the quality of daily life. Although the precise treatment of respiratory diseases has made a great progress [7–9], the pathogenesis of them still needs further elucidation.

Innate together with adaptive immunity, as natural systematic defensive barrier, is composed of immune organs, cells, and cytokines [10, 11]. The innate immunity is a natural defense that shapes in the process of long-time biological evolution [12, 13]. As the first barrier to defend infection, innate immunity participates in the resistance to pathogenic invasion and the clearance of aging, injured and even mutant cells nonspecifically. Innate immunity was firstly reported in the development of immunology and was becoming the focus of immunological research in recent twenty years, especially after the discovery of various kinds of pattern recognition receptors (PRRs) and innate lymphoid cells (ILCs) [14–17]. When the exogenous threats cannot be removed by innate immunity successfully, adaptive immunity will take part in the important defensive battle. Adaptive immunity system including humoral and cellular immunity often plays a leading role in the final clearance of invasive pathogens. The executors are T lymphoid cells and B lymphoid cells, who can both recognize antigens specifically. Different immune cells can exert protective and defensive effect synergistically with the help of multiple cytokines and protein molecules. The mechanism of adaptive immunity has been being gradually clarified since the birth of immunology. Various monoclonal antibody medicines related with adaptive immunity such as rituximab and infliximab have brought a wonderful curative effect in many refractory diseases including respiratory diseases [18, 19].

Traditionally, neutrophils, originating in bone marrow stem cells, had only been considered as a kind of innate immune cell [20]. As an essential component of innate immunity, neutrophils play an important role in killing pathogens and removing cellular debris [21]. The migration and activation of neutrophils could cause inflammation and sensitization directly or indirectly. Inflammation caused by self-immune system is really important for the solution of infection and clearance of pathogens. But the persistent inflammation in respiratory system frequently leads to some adverse diseases such as asthma, COPD, and pulmonary fibrosis. In addition, neutrophils can synergize with lymphocytes and other granulocytes, such as Th2/Th17 and eosinophils, to participate in not only innate but also adaptive immune process and promote airway inflammation [22, 23]. The interaction between neutrophils and other immune cells, endogenous composition, and foreign matter is very complex and being clarified thoroughly.

There have been more and more studies on the role of neutrophils in respiratory diseases. Recently, exosomes, neutrophil extracellular traps (NETs), deriving from neutrophil and the higher autophagy of neutrophils have been reported in multiple respiratory diseases [24, 25]. Despite that the pathogeneses of respiratory diseases are being studied extensively, there is still a long way to go to clarify the complexity and heterogeneity, especially the participation of various immune components in the development of respiratory diseases. In this review, the latest progress of neutrophils in respiratory disease such as asthma, COPD, pneumonia, lung cancer, pulmonary fibrosis, bronchitis, and bronchiolitis will be summarized.

## 2. Asthmatic Neutrophils

### 2.1. What Is Neutrophilic Asthma?

As Global Initial for Asthma (GINA, updated in 2017) elucidated [26], asthma is a heterogeneous disease, always characterized by expiratory airflow limitation and chronic inflammation. Asthma is usually categorized as different phenotypes and endotypes according to its different clinical characteristics and distinct pathological mechanism. Traditionally, asthma was classified as four different phenotypes [27], eosinophilic, neutrophilic, mixed granulocytic asthma, and paucicellular asthma according to the cellular counts of sputum, bronchoalveolar lavage fluid (BALF), or peripheral blood [28]. For example, Jodie et al. distinguished asthmatics with neutrophil proportion in sputum over 61% as neutrophilic asthma [29]. However, more and more researches have demonstrated the instability of asthma phenotypes [30–32]. Neutrophil as an essential granulocyte has been reported by many investigators to play a critical role in many immunity-associated diseases including asthma, especially steroid-refractory severe asthma [33, 34]. High blood neutrophils counts are associated with an increased risk of moderate, but not severe asthma exacerbation [35]. At the same time, the neutrophil-predominant asthmatics also tend to show a lower bronchial lability [36].

### 2.2. How Neutrophils Participate in Asthma?

#### 2.2.1. Chemotactic Activity of Neutrophils

Lower respiratory tracts used to be considered as sterile. But more and more evidence had already showed us the conflicted results [37]. *Moraxella catarrhalis* or a member of the Haemophilus or Streptococcus genera was discovered colonizing in the lower airways of asthmatics [38]. These species' colonization was associated with more differential sputum neutrophil counts and worse clinical disease status. The altered colonization would participate in the development of asthma phenotype. Infection of *H. influenza* could synergize with allergic airway diseases to induce Th17 immune responses that drive the development of neutrophilic asthma. The process above is mediated by IL-17 responses [39]. In addition, subclinical infection likely contributes to neutrophilic inflammation in airways [40].

Microbial components, which contain LPS and *β*-glucan, could synergistically cause neutrophilic asthma mediated by TLR-4 and dectin-1 [41], whose deficiency could significantly attenuate the recruitment of neutrophils induced by house dust mite (HDM) into airways [42]. Blood neutrophils from allergic asthma also show the chemotactic and phagocytic activities towards LPS and asthmatic serum [43]. Asthmatics challenged with inhaled *Dermatophagoides pteronyssinus* (DP) would promote the production of neutrophil chemotaxis [44]. Siew et al. demonstrated that neutrophil chemotaxis induced by smoke and other environmental stimulations could also be helpful for developing inflammation in airways [45].

As described above, smoking and other infectious factors can cause the accumulation of neutrophils in BALF. This is associated with the activation of phosphatidylinositol 3-kinase (PI3K) signal [46, 47]. Phosphatidylinositol 3-kinases (PI3Ks), as the key elements in the signaling cascades, play an important role in the chemotaxis of neutrophils [48]. In particular, PI3K*δ* and PI3K*γ* isoforms contribute to inflammatory cell recruitment and subsequent activation [49]. The traditional role of different PI3K isoforms in the chemotaxis of neutrophils had already been reviewed previously [50, 51]. PI3K*γ* deficiency could significantly reduce the influx of neutrophils into BALF [52]. PI3K*δ* inhibition may also prevent recruitment of neutrophils [53]. The PI3K-related inflammation and steroid insensitivity should partly be attributed to microRNA-21/PI3K/histone deacetylase 2 (HDAC2) axis as Kim et al. reported [54]. In addition, the activation of PI3K is accompanied with the release of all kinds of chemokines and cytokines, such as IL-6 and IL-8, which are related with the increased chemotactic activity of neutrophils towards the inflamed sites [55, 56]. Not only the chemotactic activity of neutrophils but also the concrete details of neutrophil activation mechanism have been making progress in recent years.

#### 2.2.2. Immune Interaction Related with Neutrophil Activation

**Figure 1:**
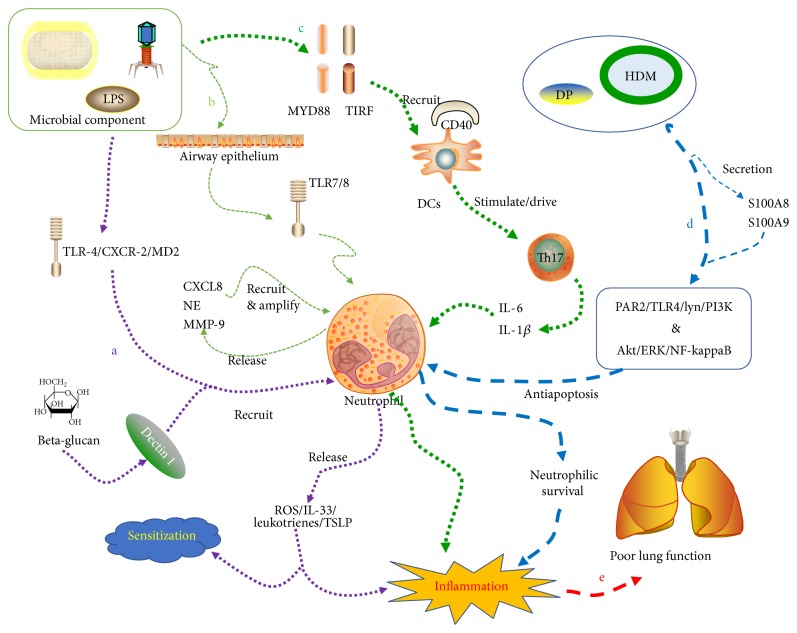
a: microbial component and environmental allergens such as LPS and *β*-glucan could recruit neutrophils via TLR-4/CXCR-2/MD2 and dectin-1. The recruited neutrophils could produce ROS/leukotrienes/IL-33/TSLP and cause inflammation or sensitization. b: the foreign allergens can act on airway epithelium and induce neutrophils to release CXCL8/NE/MMP-9, which is mediated by TLR-7/8. The released substance could amplify the recruitment of neutrophils in a positive feedback manner. c: microbial products and inhaled allergens can induce Th17 activated through TIRF and MYD88 by activating IRF-3, inducing I interferons, upregulating CD40 on DC, and finally releasing IL-6 and IL-1*β* to act towards airway neutrophils. TIRF also contribute to Th17 responses to inhaled allergens by increasing recruitment of DCs and to drive Th17 cell differentiation. d: HDM-DP could induce the secretion of S100A8/S100A9. The combination of S100A8/S100A9 might activate TLR4/Lyn/PI3K/Akt/ERK/NF-kappaB pathway to produce an antiapoptotic effect on neutrophils. e: the stimulated or survival neutrophils can cause the persistence of inflammation. As a result, the lung function is getting poorer and poorer.

As previously reviewed [57], TIR-domain-containing adapter-inducing interferon-beta (TRIF) played a key role in the induction of inflammatory mediators which could contribute to antiviral innate immune. Hsia et al. discovered that TRIF, as an essential component together with MYD88, could mediate microbial products (inhaled allergen) to induce Th17 activated. The process may include an activation of IRF-3, induction of I interferons, upregulation of CD40 on DCs or CD40L on T cells, and finally, the release of IL-6 and IL-1*β*, which are required for airway neutrophils [58]. The environmental stimulations could promote the production of IL-17. The expression level of IL-17 is also correlated with the expression of IL-8 and neutrophil numbers [45]. It was revealed that TRIF-CD40-Th17 axis participated in the IL-17-associated neutrophilic asthma. In addition, TRIF might also contribute to Th17 responses to inhaled allergens by increasing the recruitment of DCs to lung with potential to drive Th17 cell differentiation [58] (Figure 1, c). Similar to that, Siew et al. discovered that CSE (cigarette smoke extract), IL-17, and aeroallergens could act on human tracheal epithelial cells and further increase IL-6 and IL-8 production [45]. CD11b + DCs could sense some molecules in HDM extract and play a key role in the induction of HDM-induced allergic airway inflammation by inducing the expression of chemokine or chemokine receptors in DCs by expressing dectin-1 [42].

Rhinovirus infection could induce bronchial mucosal neutrophilia in subjects with asthma. Airway neutrophils during infection are positively related to virus load [59]. Recently, it has been demonstrated that LPS and viral infection could also promote the release of CXCL8/NE/MMP-9 from neutrophils, which is mediated by TLR7/8. CXCL8, as a potent chemoattractant for neutrophils released by a variety of cells including neutrophils, could amplify the recruitment in a positive feedback manner [60] (Figure 1, b). Several studies have also reported that CXCL8 levels in the airways inversely correlate with FEV1 in asthmatics [38, 61]. However, N-formyl-methionyl-leucyl-phenylalanine (fMLF), a bacterial-derived protein and ligand for fMLF receptor [62] could stimulate neutrophils from asthmatics to produce CXCL8, whose production is positively correlated with FEV_1_ and FEV_1_/FVC. It showed that circulating neutrophils might be related to airflow limitation. These exteriorly conflicting reports tell us that neutrophils may be not the main source of CXCL8 in BALF [63]. In addition, Page et al. reported that challenging with German cockroach feces towards airway could migrate neutrophils into the airways and activate neutrophil cytokine production via TLR2 [64].

#### 2.2.3. Inflammation Caused by Neutrophils

The inferior management of asthma in clinic practice is attributed to the persistent uncontrolled inflammation. Hosoki et al. report that allergens, such as pollen and cat dander, recruit neutrophils in a TLR-4/CXCR-2/MD2-dependent manner to the airways. The recruited neutrophils could produce ROS/leukotrienes/IL-33/TSLP (thymic stromal lymphopoietin) (Figure 1, a). All of these could cause the airway inflammation and allergic sensitization [65]. TLR4 expressed on hematopoietic cells is critical for neutrophilic airway inflammation following LPS exposure and for Th17-driven neutrophilic responses to the HDM lysates and ovalbumin (OVA). But at airway epithelial cells, TLR4 could also participate in the eosinophilic airway inflammation [66]. As described above, challenging with inhaled DP in asthmatics would not only increase the chemotaxis of neutrophils but also promote the production of ROS and the phagocytic activity, reflecting an enhanced systemic inflammation [44]. Moreover, apoptotic neutrophils in airway tissues could undergo secondary necrosis as the cause of inflammation [67].

TLR4 and cytokines seem to participate in the inflammatory process. The function of neutrophils from asthma patients was impaired with the lower levels of IL-8, IL-1*β*, and TNF-*α* and decreased Tlr4 gene expression [68]. All of these changes would lead to increased susceptibility and severity of infections. But this was conflicted with the discovery reported by Baines et al. which claimed that noneosinophilic asthma had an elevated level of IL-8 [69]. Simpson et al. reported that clarithromycin, which had additionally been used to reduce neutrophilic inflammation in asthma, can significantly reduce airway concentrations of IL-8 and neutrophil accumulation and activation in the airways of patients with refractory asthma [70]. Corresponding to the reports above, the withdrawal of inhaled corticosteroid (ICS) can lead to an increase of neutrophils and IL-8 in sputum [71]. The distinct role of IL-8 reported by researchers can be attributed to the different clinical research group design. In detail, noneosinophilic asthma is not equal to the neutrophilic asthma completely as previously described.

Recent investigation reported that a series of secretory protein (CCSP) could significantly reduce oxidative burst activity and increase phagocytosis of neutrophils [72]. CCSP could also enter neutrophils and alter their function. Secretoglobin protein, as a sort of CCSPs, has anti-inflammatory properties. Cote et al. reported that secretoglobin 1A1A could increase neutrophil oxidative burst and phagocytosis. Neutrophilic extracellular traps (NETs) are significantly reduced by secretoglobins 1A1 and 1A1A. Their functional difference may contribute to the pathogenesis of recurrent asthma obstruction [73]. These proteins have the potential to be novel therapeutic targets in the future.

#### 2.2.4. Prosurvival of Neutrophils

Granulocytes, including eosinophils and neutrophils, have the significant capacity to evoke tissue inflammation and remodeling. The removal of these granulocytes would contribute to the resolution of airway inflammation in asthma. Tian et al. discovered that promoted apoptosis of inflammatory cells, such as eosinophils and neutrophils would be essential for the clearance of allergen-induced airway inflammation, especially for corticosteroid-insensitive neutrophilic airway inflammation [74]. However, noneosinophilic asthma shows an enhancement of blood neutrophil chemotaxis and survival [69]. Uddin et al. enumerated the apoptotic neutrophils in sputum from asthmatic patients with different disease severities and found amongst a subset of neutrophilic asthmatics (>65% PMNs) their sputum neutrophils inversely correlated with lung function (FEV1, % predicted) due to unidentified factors present in sputum [75]. HDM-DP, as an allergen from our environment, could induce the secretion of myeloid-related protein 8 (MRP8, S100A8) and MRP14 (S100A9). The combination of them might activate TLR4/Lyn/PI3K/Akt/ERK/NF-kappaB pathway so as to produce an antiapoptotic effect on neutrophils [76] (Figure 1, d). The similar discovery was also reported by Lee et al. [77]. The survival of neutrophils in BALF would bring up a poor lung function, consistent with the decline of FEV1, as Sikkeland et al. discovered [78] (Figure 1, e).

### 2.3. Where Is the Potential Targeted Therapy?

It is believed that the failure of targeting neutrophils could be attributed to an incomplete understanding of underlying mechanism of neutrophilic asthma [79]. New insights into emerging neutrophil biology and underlying mechanisms of neutrophil phenotype might come to be the evidences of precision-based medicine. ICS is still the first-line medicine for ameliorating syndromes. Coinhalation of roflumilast and fluticasone, which reduces the counts of both neutrophils and eosinophils in BALF, could significantly improve the inflammatory condition in OVA-induced mice compared with the combination of formoterol and fluticasone [80].

Manually synthetic chemical drugs have an important curative effect on various diseases all along. Simvastatin, as an effective serum cholesterol-lowering agent could reduce the percentage of neutrophil in BALF and improve airway inflammation and remodeling in obese asthma mice [81]. Tamoxifen had a direct action on equine peripheral blood neutrophils and dampened the respiratory burst production [82]. Rosiglitazone (RSG), a peroxisome proliferator-activated receptor-*γ* agonist, has been reported to attenuate airway inflammation by inhibiting the proliferation of effector T cells in a murine model of neutrophilic asthma in vivo [48]. It can also downregulate the ratio of Treg and Th17 cells, inhibit the secretion of Th2 cytokines, and further inhibit the airway inflammatory response in asthma mice effectively [83]. AZD5069 as an antagonist of CXCR2, a receptor promoting neutrophils back to the inflamed airways, could reversibly reduce circulating neutrophils' count [84]. SCH527132, a selective CXCR2 receptor antagonist, can reduce sputum neutrophils and tend to improve the Asthma Control Questionaire scores of asthma [67].

Medicine from various plants has composed a large part in health care filed. Ligustrazine [85], water extract of *Helminthostachys zeylanica* (L.) Hook [86], astragalin as an anti-inflammatory flavonoid present in persimmon leaves and green tea seeds [87], hydroethanolic extract (70%) of *M. longiflora* (HEMI) [88], bufalin [89], and cordycepin [90] could target to the neutrophils, intervene the different inflammatory signaling pathways, and improve the prognosis of asthma. Biopharmaceutical has become a new treatment for asthma in recent years. Recombinant human activated protein C (rhAPC) could attenuate HDM + LPS-induced neutrophil migration in allergic asthma [91]. Another recombination protein, recombinant human IL-4, could inhibit airway inflammation in bronchial asthma by reducing the cytokines and inflammatory cells including neutrophils [92]. Medicine from plants and targeted biopharmaceuticals has a huge potential to play a major role in future medical field.

## 3. Neutrophils in COPD

### 3.1. Neutrophils Participate in COPD

The role of neutrophils in COPD is different from that in asthma [93]. In asthma, neutrophils are important only in some relatively rare and severe subtypes. However, neutrophils seem to always play a major role in COPD. The activation of neutrophils in the lung is directly correlated with the severity of symptoms [93]. The elevated neutrophil-lymphocyte ratio (NLR) can be used as a marker in the determination of increased inflammation and early detection of potential acute exacerbations in COPD patients. [94]. Although neutrophils in COPD patients' airway mucosa play a key role in antimicrobial defense theoretically as described in present review, a high number of neutrophils in patients' lungs are believed to correlate with poor prognosis [95]. It is well known to us all that neutrophil is an important part in maintaining our host defense and responding to injury and microbial infection. The removal of neutrophils can promote a return to homeostasis after its short-lived journey from circulation to injured or infected site. As Bratton et al. described, if not being removed, the dying neutrophils may even contribute to the ongoing inflammation, tissue damage, or autoimmune diseases by disintegrating and releasing various phlogistic cargoes [96].

### 3.2. The Role of Pathogen Infection and Proteases from Neutrophil in COPD

**Figure 2:**
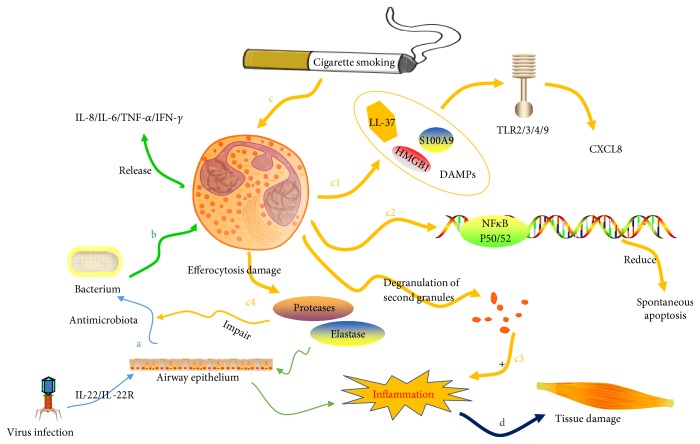
a: infection of virus can stimulate airway epithelium and produce antimicrobiota defense through IL-22/IL-22R signaling pathway. b: the colonization of bacterial pathogens could cause the release of IL-8/IL-6/TNF-*α*/IFN-*γ*. c1: cigarette smoking (CS) could induce the release of damage-associated molecular patterns (DAMPs). DAMPs can activate the innate immune system by binding to pattern recognition receptors (PRRs), such as TLR2, TLR4, and TLR9, and further induce CXCL8 release via TLR9 activation. c2: CS increase the expression of both p50 and p65 subunits of NF-kappaB in neutrophils and reduce the spontaneous apoptosis of neutrophils. c3: CS could cause the degranulation of secondary granules. This contributes to the accumulation of neutrophils and inflammation within the airways of smokers. c4: the efferocytosis damage caused by CS can increase the release of proteases and elastases. It not only impairs the antimicrobial defense but also (d) promotes the pulmonary inflammation and tissue degradation.

The pathogens' colonization, such as microbiota in lower respiratory airway, could interact with immune system. Airway bacterial loading at baseline correlates with sputum percent neutrophil count [97]. In COPD, exposure to bacterial pathogens could cause characteristic innate immune responses in peripheral blood monocytes and polymerphonuclear neutrophils (PMN), accompanied with the elevated protein expression of IL-8/IL-6/TNF-*α*/IFN-*γ* [98] (Figure 2, b). At the same time, Guiot et al. also observed that COPD patients had higher IL-6, IL-8, TNF-*α*, and MMP-9 in their induced sputum [99]. Treatment with levofloxacin in stable COPD could reduce the bacterial loading in a short time. This reduction is associated with the decrease of neutrophilic airway inflammation in patients with high airway bacterial loads [97].

Neutrophils contain a great deal of proteases, inflammatory mediators, and oxidants [100]. IL-22/IL-22R signaling pathway plays a pivotal role in antimicrobial defense. Influenza virus can promote the expression of IL-22R in human bronchial epithelia cells (Figure 2, a). Neutrophil-derived proteases may contribute to COPD by impairing the antimicrobial IL-22/IL-22R signaling pathway and decreasing the expression of antimicrobial effectors such as *β*-defensin-2. This process probably enhances pathogen replication and ultimately causes COPD exacerbations [95].

Neutrophil elastase (NE), one of the main proteases produced by neutrophils, plays an important role in inflammatory process. NE is able to increase the release of chemokines from epithelial cells with the activation of p38-*α*-MAP-kinase. The production of these chemokines can be blocked by roflumilast N-oxide combined with prostaglandin E_2_ [101]. Neutrophil elastase-generated fragments of elastin (EL-NE) is different between stable and exacerbation of COPD patients. The serum level of EL-NE is associated with lung function [102]. Elastin breakdown mediated by neutrophil elastase is associated with COPD-induced inflammation [103]. *α*-1-Antitrypsin (A1AT), as an endogenous inhibitor of NE, can limit lung damage. Its effect had been already reviewed by Meijer et al. [93]. Deficiency of it is known as a risk factor for lung function [93]. Geraghty et al. reported that bioactive A1AT could modulate phosphatase 2A, which played a key role in COPD and expressed on the neutrophils to prevent inflammatory and proteolytic responses triggered by TNF-*α* simulation in the lung [104]. Whether A1AT or its unknown analogues can be used as a potential novel medicine needs further scientific research and clinical validation.

### 3.3. Adverse Effect of Cigarette Smoke via Neutrophils

Cigarette smoking (CS), a main environmental trigger of COPD [93], can decrease sputum neutrophils significantly. Experiments in vitro showed that CS could induce necrotic neutrophil cell death through mitochondrial dysfunction, apoptosis inhibition, and damage-associated molecular pattern (DAMP) release [105, 106]. DAMPs can activate the innate immune system by binding to pattern recognition receptors (PRRs), such as TLR2, TLR4, and TLR9. DAMP signaling plays an important role in the activation of neutrophils during COPD exacerbations. Serum level of DAMP gene expression is increased during COPD exacerbations [106]. TLR2, TLR4, and NLRP3 expressions in neutrophils are increased during acute exacerbations of COPD compared with stable disease. The activation of TLR2/TLR4 can induce the activation and the migration of neutrophils and may thus contribute to the elevated airway inflammation during COPD exacerbations [107]. The detailed positive feedback loop between DAMPs and TLR4 and the function of neutrophils' TLR4/TLR3 in respiratory diseases had already been reviewed by other researchers [106–108]. The novel therapeutic strategies aimed at DAMPs and its receptors need more attention.

Normal human bronchial epithelial cells could release more CXCL-8 significantly when stimulated with the supernatants from CS-treated neutrophils [105]. While in COPD patients, it was showed that there was an increase of inflammatory response and a decrease of PMNs apoptosis, which is independent from antiapoptotic cytokines such as CXCL-8 [109]. Mortaz et al. reported that cigarette smoking extract (CSE) could induce CXCL8 release via TLR9 activation [110] (Figure 2, c1). In addition, CSE could cause the degranulation of secondary granules. This may contribute to the accumulation of neutrophils and inflammation within the airways of smokers (Figure 2, c3). Furthermore, it promotes the pulmonary inflammation and tissue degradation [111].

Many kinds of membrane moleculars expressed on the surface of neutrophils are able to take part in mediating the biological activity resulted from cigarette smoke. Hoonhorst et al. reported that young individuals susceptible to COPD showed a significantly higher increase in the expression of Fc-*γ*-RII (CD32) in its active forms (A17 and A27) on neutrophils after smoking. This may indicate that systemic inflammation could participate in the early induction phase of COPD [112]. CD9, a transmembrane protein of the tetraspanin family, facilitates some pathogens and other foreign matters. HDAC and Rac have already been demonstrated that they are required for efferocytosis [100]. Noda et al. reported that smoking could impair efferocytosis of neutrophils via inhibition of HDAC/Rac/CD9 pathways (Figure 2, c4). The following release of toxic intracellular contents from apoptotic neutrophils could cause tissue damage [100] (Figure 2, d).

The ability of ingesting respiratory pathogen is compromised in CSE-exposed neutrophils. As a result, it contributes to persistent existence of bacterium in the smokers' lungs and promotes further recruitment of neutrophils. Guzik et al. discovered a lack of apoptotic neutrophil populations in smokers' lungs, and these smokers exhibited an increased susceptibility to bacterial infections [111].

Human neutrophils share typical cell death features, including apoptosis, autophagy, and necrosis after exposure to CSE. These neutrophils could be effectively recognized and phagocytized by monoderived macrophages [111]. Neutrophils could also undergo a spontaneous and phagocytosis-induced apoptosis in a caspase-3-dependent manner. The suppression of caspase-3 activity induced by CSE does not alter spontaneous apoptosis but impair the phagocytic activity. The complex functions of caspase-3 may contribute to the persistent existence of neutrophils in smokers' lungs. At the same time, caspase-3 could also cause a higher incidence of community-acquired pneumonia [113]. In addition, percentage of sputum neutrophils undergoing spontaneous apoptosis in the subjects with COPD reduced significantly. The increased expression of both p50 and p65 subunits of NF-kappaB in neutrophils from COPD individuals may explain the phenomenon above [114] (Figure 2, c2). Interestingly, Makris et al. observed an increase of sputum apoptotic neutrophils' percentage in ex-smoking patients with COPD by the way of in situ detection with TUNEL assay technique [115]. Whether these conflict results are related with the distinct exclusion criteria of subjects needs further investigation.

### 3.4. Study on the Extracellular Traps of Neutrophils

Polymorphonuclear neutrophils have attracted new attention due to its ability of releasing web-like extracellular structures, which has been named as neutrophil extracellular traps (NETs) recently [116]. NETs' formation has been identified to be an essential part of innate immunity [117]. These NETs deriving from nuclear chromatin may have an ambiguous two-side effect on antimicrobial defense and host tissue damage. Microbicidal and cytotoxic proteins decorate the NETs, which are constituted with DNA strands of varying thickness. Their principal chemical structures have been characterized at molecular and ultrastructural levels in recent years. Nonetheless, many features relevant with cytotoxicity are still not clear completely [116]. Pedersen et al. observed a significant upregulation of NET formation, which was associated with significantly higher concentration of extracellular DNA in sputum supernatant of COPD [112]. Astrid et al. studied the genesis and structure of NETs from sputum of COPD patients. It was concluded that the genesis of NET was an integral part of COPD pathology. Moreover, the release of “beads-on-a-string” DNA studded with noncitrullinated histones, as Astrid et al. described, is a common feature of genesis of NETs in vivo. All of these are relevant to the antimicrobial and cytotoxic effects of NETs [116].

### 3.5. Pharmacological Mechanism of Neutrophil Targeted Therapy in COPD

Bronchodilators have always been a clinical first-line medication to ameliorate the symptoms in COPD [93]. Anderson et al. tested the effect of three *β*2-agonists (formoterol, indacaterol, and salbutamol) on the inhibition to the proinflammatory activity. The results showed that formoterol and indacaterol could effectively decrease the potential harm produced by stimulated neutrophils in vitro, while the effect of salbutamol was weaker. The anti-inflammatory actions of formoterol and indacaterol could contribute to the therapeutics in COPD [118]. Tanabe et al. reported that thioredoxin-1 could improve neutrophilic inflammation by suppressing the release of GM-CSF and enhancing the expression of MAP kinase phosphatase 1. All these indicate that thioredoxin-1 is a novel potential therapeutic agent for treating the exacerbation of COPD [119]. Keratin sulfate disaccharide repeating unit designated L4 could significantly attenuate inflammation in the lung by reducing neutrophil influx as well as the levels of inflammatory cytokines, tissue-degrading enzymes (matrix metalloproteinases), and myeloperoxidase in BALF [120]. More clinical trials are needed to further reveal the potential of these related medicines.

Similar to asthma, ICSs are also the common therapy strategy in clinical practices to control COPD. However, the discontinuation of ICS can increase the inflammation in COPD showing as an increased sputum cell counts including neutrophils [121]. At the same time, the effect of ICS in COPD patients is also dependent on the genotype of glucocorticoid-induced transcript 1 rs37973 in COPD patients. The effect of dexamethasone-mediated neutrophil apoptosis is impaired in homozygous GG genotype. In addition, CSE-induced neutrophil apoptosis could only be slightly attenuated by a relatively higher concentration GG genotype. As for AA and AG genotypes, dexamethasone can reduce the proapoptotic effect of CSE in a concentration-dependent manner [122]. Besides, aminophylline and theophylline, two typical medicines used in therapy for COPD, could restore the impairment caused by trichostatin A (TSA), a Rac inhibitor. This protective effect may have the potential to develop a novel therapeutic strategy to restore efferocytosis in COPD patients [100].

## 4. Neutrophils in Infectious Pneumonia

Pneumonia is a common infectious lung disease accompanied with inflammation in airway and alveolar and interstitial lung. Pneumonia is usually categorized as community-acquired pneumonia (CAP) and hospital-acquired pneumonia (HAP). Bacterial infection is the most common etiology of pneumonia as observed in our clinical practice. Neutrophils can function with other immune cells synergistically to control the pathogenic infection in pneumonia [123]. Evidence on the participation of neutrophil in the pathogenesis of pneumonia has been accumulated in recent years. For example, the neutrophil percentage in BALF was higher in the relapse group of organizing pneumonias and may also be considered as a predictive factor of organizing pneumonia relapse as Onishi et al. reported [124]. But, on the contrary, CAP had significant low neutrophil counts in peripheral blood [125]. In addition, NLR together with red blood distribution can even be used as adjuncts to distinguish HAP and CAP [126].

### 4.1. *Pseudomonas aeruginosa* Pneumonia

*Pseudomonas aeruginosa* (PA) as an important opportunistic human pathogen can take part in the pathogenesis of pneumonia. PA lives in biofilm-like cell and aggregates at sites of chronic infection. During growth in a biofilm, *P. aeruginosa* dramatically increases the production of filamentous Pf bacteriophage (Pf phage). The Pf phage can trap within the lung PA by preventing the dissemination of *P. aeruginosa* from the lung in pneumonia and inhibit bacterial invasion of airway epithelial cultures. Importantly, the production of Pf phage was also associated with reduced neutrophil recruitment [127].

Mechanical ventilation is routinely used to treat patients with respiratory distress. However, a number of patients on ventilators exhibit enhanced susceptibility to infections and develop ventilator-associated pneumonia (VAP). PA is one of the most common species of bacteria found in these patients [128]. Mechanical ventilation after supernatant from PA-stimulated macrophages can induce more neutrophil sequestration in the lungs in wild-type mice than JNK1-deficient mice. Moreover, the pathogenesis mechanism of PA-VAP may involve the production of TNF-*α* through activation of IKK/NF-kappaB pathways in alveolar macrophages and JNK signaling pathway in the lungs [129]. A recent research indicates that IRF-3 can exacerbate PA-induced mortality in mice by inhibiting neutrophil adhesion and recruitment to the lungs [130]. In addition, the inhibition of full-length receptor for advanced glycation end product (FL-RAGE) shedding can be a novel mechanism for controlling inflammation to acute PA pneumonia [131]. Deepening the exploration of its pathogenesis will contribute to the next future clinical application.

The persistent presence of PA and huge recruitment of neutrophils in the lung are associated with the elevated level of high mobility group box 1 (HMGB1) in airways in respiratory diseases [132]. Exposure to hyperoxia leads to a significant elevation in HMGB1 and increased mortality in C57BL/6 mice infected with PA. Treatment of these mice with a neutralizing anti-HMGB1 monoclonal antibody can result in a reduction in bacterial counts, injury, and numbers of neutrophils in the lungs and an increase in leukocyte phagocytic activity [133]. This finding reveals a potential medical target. More related clinical trials are needed to validate it.

### 4.2. *Streptococcus pneumoniae* Pneumonia

*Streptococcus pneumoniae* (SP) is a common cause of pneumonia and infective exacerbations of chronic lung disease [134]. During lung infection, mice colonized with SP had an increased early neutrophil recruitment and reduced bacterial colony-forming units in the lungs and BALF. Colonization-induced protection was lost when experiments were repeated in B-cell-deficient or neutrophil-deficient mice [134]. Yet how neutrophils specifically prevent SP lung infection has been complex and still unclear till now.

Nucleotide-binding oligomerization domain-containing (NOD) 2 as a pattern recognition receptor can detect peptidoglycan fragments of SP. Nod2-deficient blood neutrophils displayed a reduced capacity to internalize pneumococci in vitro. But NOD2 does not contribute to host defense during pneumococcal pneumonia. Pneumococcal capsule can impair recognition of SP by NOD2 as Hommes et al. discovered [135]. Triggering receptor expressed on myeloid cells-1 (TREM-1) is a receptor on phagocytes known to amplify TLR- and NOD-like receptor inflammatory signaling [136, 137]. TREM-1/3 deficiency leads to an increased lethality, accompanied by enhanced growth and dissemination of SP. Trem-1/3-deficient mice demonstrated a strongly impaired innate immune response in the airways reflecting as a delayed influx of neutrophils [138]. However, the influx of endothelial protein C receptor positive neutrophils into lung tissue can cause pneumonia after the infection of SP, because endothelial protein C receptor can impair antibacterial defense in pneumococcal pneumonia [139].

Moreover, IL-1 can contribute to the host defense against SP independent on the recruitment and the bacteria-killing ability of neutrophils [140]. Alveolar neutrophils with single immunoglobulin IL-1 receptor-related molecule (SIGIRR) deficiency exhibit an increased capacity to phagocytose viable pneumococci but no impact on neutrophil recruitment. Besides, SIGIRR as a negative regulator of TLR signaling can impair the antibacterial host defense during pneumonia caused by SP [141].

### 4.3. *Staphylococcus aureus* Pneumonia

*Staphylococcus aureus* (SA) is a Gram-positive bacterium that persistently colonizes about 20% of the human population [142]. This high prevalence of SA is responsible for various illnesses in humans and animals worldwide, including the respiratory diseases [143, 144]. Similar to SP, infection with SA can exhibit an early increase in neutrophils that did not persist despite continued presence of the bacteria in neonatal mice. However, adult mice exhibited an increase in neutrophil recruitment that coincided with reduced bacterial titers [145]. SA, as an important pathogen, can efficiently cleave the pulmonary surfactant protein-A (SP-A), a major component of immune functions during SA infections. This degradation appears to result in a decrease or complete abolishment of SP-A biological activity, including the promotion of SA phagocytosis by neutrophils [146]. Recently, Dietert et al. reported that in SA pneumonia murine model, the deficiency of calcium-activated chloride channel regulators (mCLCA3) can lead to a decrease of neutrophil infiltration during infection. Moreover, mCLCA3 appears mainly to modulate leukocyte response via IL-17 and murine CXCL-8 homologs in acute SA pneumonia [147].

### 4.4. *Klebsiella pneumoniae* Pneumonia

Nosocomial infection with *Klebsiella pneumoniae* (KP) is a frequent cause of gram-negative bacterial sepsis [148]. C-type lectin receptor is an innate immunity-related receptor, which can interact with pathogenic-associated molecular patterns [149]. Mincle and macrophage galactose-type lectin-1 (MGL1) are C-type lectin receptors. The deficiency of them can lead to a massive accumulation of neutrophils and a severe hyperinflammation in the lungs of KP-infected pneumonia. Importantly, Mincle-deficient neutrophils had an impaired ability to phagocytize bacteria and to form extracellular traps (NETs), which could clear the invading KP [148]. Similarly, MGL1-deficient neutrophils exhibited an increased influx in pneumonic lungs of KP-infected mice. Neutrophilic inflammation resolution relies on MGL1 during KP infection [150]. As previously described, NLRC4 belongs to the NOD-like receptor family and is involved in the assembly of the inflammasome complex [151]. NLRC4 can participate in the neutrophil chemoattractant in the lungs infected by KP. NLRC4 signaling contributes to KP-induced lung inflammation and neutrophil accumulation, which can be partially rescued by exogenous IL-1*β* in the lungs of NLRC4-deficient mice [151]. Myeloid-related protein 8 (MRP8, S100A8) and MRP14 (S100A9) are the most abundant cytoplasmic proteins in neutrophils. MRP8/14 heterodimers can inhibit bacterial dissemination and prevent the growth of KP in vitro. Mrp14 can take part in the genesis of neutrophils NETs to inhibit KP growth. Taken together, MRP8/14 is a key player in protective innate immunity during KP pneumonia [152].

### 4.5. *Mycoplasma pneumoniae* Pneumonia and Recent Therapy Advances in Pneumonia

*Mycoplasma pneumoniae* is also a significant cause of respiratory diseases including CAP for all ages [153]. *Mycoplasma pneumoniae* pneumonia may be altered by the level of host cell-mediated immunity [154]. *Mycoplasma pneumoniae* pneumonia has an increase of neutrophils and IL-6, especially in severe group. Compared to acute stage, a decreased percentage of neutrophils and IL-6 level was observed at the recovery stage in children with severe *Mycoplasma pneumoniae* pneumonia [155]. However, treatment with prednisolone or cyclosporin-A leads to marked neutrophils and exudates in the alveolar lumen.

Some traditional antibiotic therapeutic strategies such as macrolide containing regimen showed no statistical difference between cytokine levels or neutrophil activity for CAP patient [156]. Targeted and precise therapy has made an advanced progress in recent years. Vaccine as a typical biological therapy strategy had been attracting a huge attention since it was created. For example, recombinant Bacillus Calmette-Guerin (BCG) vaccine can decrease the infiltration of neutrophils within airways and reduce the viral loads in BALF in mice infected with respiratory syncytial virus (RSV) showing a potential to prevent pneumonia [157]. Trivalent pneumococcal protein recombinant vaccine vaccination results in a reduction in SP-induced lethality, enhanced early clearance of SP in lungs due to more rapid and thorough phagocytosis of SP by neutrophils, and correspondingly, a reduction in lung inflammation and tissue damage [158]. In addition, cathelin-related antimicrobial peptide appears to be protective in models of pneumonia [159]. *α*-Tocopherol form of vitamin E can reverse age-associated susceptibility to SP lung infection by decreasing pulmonary neutrophil recruitment [160].

## 5. Neutrophils Participate in Other Respiratory Diseases

### 5.1. Neutrophils in Lung Cancer

Lung cancer is still a common killer in all kinds of cancers and is also one of the most common cancers diagnosed globally [161]. The traditional role of neutrophils in pathogenesis of many kinds of tumors had already been reviewed by Zhang et al. briefly [162]. Here, we focus on the recent advances about the participation of neutrophils in lung cancer. It has been demonstrated that neutrophils could participate in the carcinogenesis in murine lung cancer model [163]. Lung squamous cell carcinoma mouse models contain more tumor-associated neutrophils compared to mouse adenocarcinomas [164]. Elastase from neutrophils could involve in lung cancer by inducing mitogenesis after entering the cells [165]. BALF from lung cancer patients contained higher level of neutrophils and lower percentage of total macrophages [166]. BALF of lung cancer patients had markedly higher levels of VEGF and IL-8, which was positively correlated to the numbers of neutrophils and lymphocytes. Tumor-associated neutrophils represent an important source of MMP-9, whose expression in tumor region is increased in non-small-cell lung cancer [167]. According to their results, the detection of infiltrating inflammatory cells and proangiogenic factors have the potential to be diagnosis indexes for cancerous inflammation in lungs [166].

Neutrophil-lymphocyte ratio (NLR), reflecting host immunity and systemic inflammation that facilitates tumor growth, could be an independent prognostic index for lung adenocarcinoma patients who undergo the complete resection [168]. NLR can also be an independent prognostic factor for overall survival. The evaluation of NLR can help identify patients with poor prognosis and appears to be a useful prognostic marker in clinical practices [169]. The model established by Jiang et al. utilizing multiple immunological markers, such as monocyte ratio, NLR, PD-L1 immunostaining score and PD-1-positive stained tumor-infiltrating lymphocyte counts, can offer a novel tool for survival prediction. This model has important clinical implications for patients with squamous non-small-cell lung cancer [170]. At the same time, NLR, together with other parameters such as age, gender, and smoking history, can be used to predict the prognosis of small cell lung cancer [171]. Consistent with the findings from Jeong et al. [172], Derman et al. found that the progressive increases of NLR are associated with the progressive disease, inferior overall survival, and weight loss in non-small-cell lung cancer patients [173]. The precise identification and prediction to the prognosis of all lung cancers with different histotypes is beneficial for future individualized therapy [170].

More recently, the role of myelomonocytic siglecs has attracted a greater attention [174, 175]. It is a receptor engaging lineage with sialic acids with dual functions towards cancer progression depending on the different stages of tumor growth and the microenvironment. Neutrophils involved in this process play an important role. Neutrophils can express siglec-9 and interact with tumors. Tumors could interact and suppress the activation of neutrophils utilizing the ligand expressed on the tumor cells and acting on the siglec-9. In keeping with this, human polymorphism of the related gene that reduced siglec-9 binding to carcinomas could improve the survival of patients with early non-small-cell lung cancer [176], which accounts for 85 percent of all lung cancer according to their histotypes [177]. As for lung cancer histotypes, it has been demonstrated that lung cancer could exhibit a pronounced heterogeneity and differential immunological characteristics. For example, adenosquamous carcinoma showed a histotype-specific recruitment of CD11b^+^Gr-1^+^ tumor-associated neutrophils [178].

Metastases are the major cause of death from cancer. Lung is the most common metastatic site for many other cancers [179]. Impaired type I IFN signaling could develop more lung metastases. The higher metastasis is accompanied with massive neutrophil accumulation in the lungs. This is most likely due to elevated G-CSF levels in serum and enhanced CXCR2 expression on neutrophils. Reduced neutrophilic cytotoxicity against tumor cells can enhance metastasis [179]. Lung-infiltrating neutrophils facilitate an improved premetastatic niche formation [180–182]. Developing premetastatic niche can enhance metastasis [179]. In addition, intranasal delivery of CCL2 increases CD4+ T cell recruitment to the premetastatic niche of the lung, and this correlates with enhanced seeding and growth of tumor cells [183], while CCL2 shows a potential antitumor activity in tumor-entrained neutrophil-mediated tumor killing in vitro. In addition, *γδ*-T-cell could indirectly act on systemic expansion and polarization, suppress CD8+ T cell, and then cause a sequent metastasis formation. All of these indicate that the interaction between *γδ*-T-cell and neutrophil may contribute to the metastasis of carcinoma [184].

There are some interesting researches about how neutrophils participate into the pathogenesis of metastatic lung cancer. Bald et al. reported that ultraviolet (UV) exposure of primary cutaneous melanomas could promote metastasis dependent on the recruitment and activation of neutrophils which is initialed by HMGB1 [185]. It is also reported that an inhibitory host protein member of B7 family called as B7x may promote cancer cells to metastasize through interacting with innate and adaptive immune systems. The presence of B7x is correlated with an increased infiltration of tumor-associated neutrophils into tumor-bearing lungs [186]. Colorectal cancer can process a lung metastasis, which is dependent on CCL15-CCR1 axis. Their immunofluorescent staining results showed that most CCR1+ cells around lung metastases were tumor-associated neutrophil [167]. Although the role of neutrophils in metastatic lung cancer is still unidentified clearly, more and more emerging researches will tell a systematic integral story in the future.

### 5.2. Neutrophils in Pulmonary Fibrosis and Cystic Fibrosis

Pulmonary fibrosis is a common interstitial lung disease [187]. Traditionally, pulmonary fibrosis is tightly associated with immune component in the lung. A greater number of neutrophils in the BALF were associated with the increased early mortality of pulmonary fibrosis [188]. The end-stage cystic fibrosis (CF) explant lung tissue showed an increase of neutrophils. At the same time, there was a disproportionate increase of neutrophils around the airway in CF [189].

Pulmonary fibrosis murine model is always established with bleomycin injection. Neutrophils can take part in the acute inflammatory process. The occurrence of the acute inflammation is accompanied with the production of collagen in parallel [190]. Interestingly, bleomycin-induced lung fibrosis can be relieved by the reduction of soluble glycosaminoglycan (GAG), which could reduce the neutrophil transmigration and decrease the CXCL-8/neutrophil-mediated inflammation [191]. Carbohydrate antigen sialyl Lewis is secreted from the bronchial gland apically. Obayashi et al. reported that carbohydrate antigen sialyl Lewis in BALF could participate in the process of lung injury and repair in pulmonary fibrosis by modifying the function of neutrophils [192].

Serum amyloid P (SAP) is a pattern recognition molecule and could interact with pathogens and cell debris to promote their removal by macrophages and neutrophils [193]. Cox et al. reported that SAP could strongly affect several aspects of innate immune system and reduce fibrosis by binding to SAP-binding receptor (DC-SIGN), which is present on mouse lung epithelial cells. Binding of DC-SIGN receptor with SAP could reduce neutrophil accumulation in the acute lung inflammatory model and alleviate pulmonary fibrosis by increasing levels of immunosuppressant IL-10 [194]. In addition, they also reported that SAP could inhibit fibrocyte differentiation and reduce neutrophil adhesion by binding to Fc-*γ*-RI on monocytes and binding to Fc-*γ*-RIIa on neutrophils, respectively [195].

Cystic fibrosis (CF) lung disease as a genetic disease is displayed as a chronic and nonresolving activation of innate immune system, accompanied with the release of neutrophil-derived oxidants and proteases and chemokines and an infiltration of neutrophils into the airways [196]. Subjects with stable CF had not only significant elevated levels of proinflammatory genes and its products but also an elevated MMP8/9 and neutrophil elastase [197]. In addition, patients with CF and small airway disease had pronounced sputum neutrophil counts and elevated level of IL-6 [198]. The traditional role of immunity in CF had already been reviewed by Rieber et al. [196]. PMNs, which is recruited massively into the cystic fibrosis lumen, could modulate arginase 1 and suppress the early PMN-driven T cell in CF. All of these might hamper the resolution of infection and inflammation in CF airway lumen [199].

HMGB1 is an alarmin released from macrophages after infection or inflammation and is a biomarker of lung disease progression in patients with cystic fibrosis [200]. Entezari et al. demonstrated that the elevated levels of HMGB1 in CF airways were essential for neutrophil recruitment and persistence of PA in the lung, which could significantly contribute to mortality in cystic fibrosis [132]. The infection of PA can secrete epoxide hydrolase, a kind of CF transmembrane conductance regulator (CFTR) inhibitor, which could cause neutrophil activation and tissue inflammation. The hydrolase could also increase IL-8 concentration, which drives neutrophils' transepithelial migration in vitro as illustrated above. Finally, the lung function of CF patients is impaired [201].

CF is a fatal recessive genetic disease. Ng et al. demonstrated that CF could be attributed to the mutations in the CFTR gene. In detail, the mutation of the gene could compromise the phagocytic capacity of neutrophils and contribute to the infection of the CF lung [202]. Recently, Duchesneau et al. found that bone marrow cell delivery therapy can contribute to the restoration of CFTR expression in airway epithelium by recruiting neutrophils and macrophages [203].

Moreover, other related gene polymorphisms or the difference of expressive level can also have effect on the pathogenesis of CF or other lung fibrotic diseases. Hector et al. demonstrated that interferon-related development regulator-1(IFRD1) expression of neutrophils was systemically upregulated in CF. This regulation was related to the production of ROS and was modulated by chemokines in airway fluids, such as CXCL-8 and CXCL-2. The decrease of lung function was associated with the genotype of IFRD1 [204]. Forkhead transcription factor 3 (Foxp3) is a critical regulator of Treg [205]. The overexpression of Foxp3 in radiation-induced lung inflammation also showed a significant inhibition of neutrophilic infiltration in BALF. At the same time, overexpression of Foxp3 can decrease the expression of inflammatory and fibrosis-related genes [206]. Extracellular superoxide dismutase 3 (SOD3) is the only extracellular enzymatic defense against the free radical, superoxide. Impaired SOD3 activity is implicated in inflammatory and fibrotic lung and vascular diseases as Mouradian et al. reviewed. However, the redistribution of superoxide dismutase 3 as a result of R213G single-nucleotide polymorphism could protect mice from bleomycin-induced fibrosis by resolving the neutrophil infiltration in BALF [207].

The potential therapy of pulmonary fibrosis is rarely reported in recent years. Recently, Yang et al. found that glaucocalyxin A (GLA) could exert antipulmonary fibrosis activity in mice. GLA could significantly improve survival in bleomycin-treated mice and reduce the weight loss caused by fibrosis. At the same time, GLA could alleviate the infiltration of neutrophils in the lungs and attenuate the increase of proinflammatory cytokines in lung tissue and BLAF. In addition, GLA could inhibit the activation of NF-kappaB in fibrotic lungs [208]. Acebilustat, as a potential leukotriene A4 hydrolase inhibitor, could reduce sputum neutrophil counts by 65% in CF patients treated with 100 mg dosage [209]. Targeting chemotaxis of neutrophils has been a promising therapeutic direction [210]. Intracellular secretory leukoprotease inhibitor can exert an anti-inflammatory effect on neutrophils of individuals with CF and COPD by inhibiting the excessive influx of neutrophil [211]. PA401 as a recombinant therapeutic protein can disrupt the CXCL8:GAG complexes. And then, the chemokine CXCL8 is degraded. As a result, the chemotaxis of neutrophil and the inflammation decreased [212]. All in all, with the development of the detailed pathogenesis of pulmonary fibrosis, the targeted modulation of the related pathways may be of therapeutic benefit to patients.

### 5.3. Neutrophils in Bronchitis and Bronchiolitis

Acute and chronic bronchitis and bronchiolitis are common respiratory diseases [213]. Despite that most patients have a good prognosis, a few people still may be persistently unhealed. Immunity plays a critical but unidentified role in this process. The related inflammatory mechanism of neutrophils in airway inflammatory diseases and the role of proteases, mediators, and TLR2 in the incidence of the illness had already been reviewed [214]. But science has never stopped unraveling its nature. Recently, it has been demonstrated that neutrophilic infiltration in nasopharyngeal aspirate (NPA) samples was positively correlated with the degree of airway tissue injury in infants hospitalized with acute bronchiolitis [215].

Increased level of IL-8, a potent neutrophilic chemokine, is often strongly correlated with an increase in cellular infiltration [215]. For example, Dixon et al. reported that breastfed infants had lower level of IL-8 in their nasal compared to the formula-fed controls hospitalized with severe bronchiolitis. Meanwhile, there is a decrease in cellular infiltration, whose predominance is mature, secondary granule-laden neutrophils [216]. Chronic bronchitis is a risk factor for COPD. Patients with chronic bronchitis and COPD exhibit reduced immune regulation and increased innate immunity response in the lung [217]. Sahlander et al. have observed an increase of blood neutrophils in farmers exposed to the organic material. These farmers often suffered from the chronic bronchitis [218]. But in a more recent experiment, they only found that both the expression of CD62L and CD162 on blood neutrophils and the expression of CD14 on sputum neutrophils decreased. All of these indicated that chronic exposure of organic material may participate in the pathogenesis of chronic airway diseases, such as chronic bronchitis. This may involve the participation of neutrophils. In addition, they also discovered the exposure could increase the presence of bacteria in airways [219].

The presence of potentially pathogenic bacteria is positively correlated the severity of bronchiolitis. The percentage of neutrophils is higher in patients with potentially pathogenic bacteria [220]. Protracted bacterial bronchitis also had a marked neutrophil infiltration and more respiratory bacterial pathogens load, especially *Haemophilus influenza*. This may be related with activated innate immunity [221]. PA is very common in respiratory airways. Club cell secretory protein (CCSP) is a regulator, which could exert an immunosuppressive, anti-inflammatory, antiproteinase, and antiphospholipase A2 activities. Chronic PA inflammation can lead to chronic bronchitis in the CCSP-deficient mice. Neutrophils are increased in the BALF from the CCSP-deficient mice in comparison to wild-type mice [222]. In addition, colonization with Gram-negative bacteria was associated with higher levels of proinflammatory cytokines. The colonization would increase the severity of disease [223]. Borthwick et al. reported that conditioned media from PA-infected epithelial cells induced a potent proinflammatory phenotype in fibroblasts via an IL-1*α*-/IL-1R-dependent signaling pathway. The evaluated level of IL-1*α* is significantly correlated with IL-8 and neutrophil percentage in BALF [224].

Viral etiology could potentially contribute to the pathogenesis of bronchiolitis [215]. Human respiratory syncytial virus (HRSV) infections have a close relationship with many respiratory diseases, such as bronchiolitis, asthma, and pneumonia [225]. Neutrophils together with its proteases, NETs, and cytotoxic and direct interaction with infected epithelial cell play an important role in the pathological process [225]. RSV could induce NET formation in vitro. Conversely, NETs can capture RSV virions, indicating an antiviral role [226]. Bronchiolitis, usually requiring hospitalization in infants, is caused predominantly by RSV. It is one of the main causes of infant mortality and morbidity in developed world [216]. Suarez et al. reported that infants with RSV bronchiolitis had more systemic inflammatory cells, such as neutrophils and leukocytes, and more pathogenic bacterial colonization in nasopharyngeal. Besides, neutrophil infiltration is independent on the viral etiology nor the degree of viral coinfection, providing support for the neutrophil as a target of therapeutic intervention for the treatment of bronchiolitis in all virus-positive infants as Cavallaro et al. reported [215]. It is pleasuring that BPZE1, as a kind of attenuated Bordetella pertussis vaccine, could markedly attenuate RSV by inducing the efflux of neutrophils and increasing the production of IL-17 by CD4+ T cells [227].

In a special case, patients undergoing lung transplantation often suffer from obliterative bronchiolitis (OB). Clinical research showed us that the percentage of lymphocytes and neutrophils increased and the percentage of macrophages reduced in BALF of patients with OB [228]. This is similar with the reports, which suggest an involvement of neutrophils in OB from both Vandermeulen et al. and Eckrich et al. [229, 230]. Tiriveedhi et al. reported that neutrophil can participate in the obliterative airway disease and cause the early injury after passive transfer of CD8+ T cells [231]. They also reported that antibodies to MHC class I of the transplanted lung could induce both innate and adaptive cellular immune responses, which is characterized by a predominance of Th17. Their data indicated that Treg cells could suppress “anti-MHC induced IL-8-mediated neutrophil infiltration,” which is critical for the development of obliterative airway disease [232]. VEGF-C/VEGFR-3 signaling can be involved in the pathogenesis of OB [233]. Upregulation of VEGF-C/VEGFR-3 signaling could induce epithelial activation, neutrophil chemotaxis, and significant neutrophilia. Both neutrophils and neutrophil chemoattractant human IL-8 contribute to the development of OB for its inflammatory infiltration [233].

Hyaluronan is an extracellular matrix component, which has been demonstrated to activate innate immunity, regulate inflammation, and could accumulate in BALF and blood of lung transplant recipients with OB. The low-molecular-weight form of hyaluronan can abolish the tolerance and promote the rejection of lung transplant through a mechanism dependent on innate immunity and neutrophils [234]. More details about its mechanism need further illustration. Adenosine is produced to protect tissues from injury when ischemia or inflammation occurs [235]. Zhao et al. described the role of A_2B_R, a receptor of adenosine in the pathogenesis of OB. Neutrophil infiltration was decreased in A_2B_R knock-out OB model on day 3 and day 21 but increased in wild-type models in the same time point. The results showed us that neutrophils could also take part in the pathogenesis of OB depending on A_2B_R to some extent [235]. In contrast, A_2A_R could also participate in the inflammatory process in OB. Neutrophil infiltration was increased in A_2A_R knock-out OB model [236]. Whether the exogenous pharmaceuticals of adenosine can be helpful to relieving OB needs future clinical validation.

## 6. Conclusions

The critical role of neutrophils in immunity-associated diseases including respiratory diseases cannot be overlooked. The colonization of microbiota in airway acts as a trigger of neutrophilic inflammation. Different stimulations from our environment could produce a chemotactic activity towards the inflammatory sites for neutrophils. Innate and adaptive immune component could participate in the activation of neutrophils in many different respiratory diseases. Neutrophils often cooperate with lymphocytes synergistically and constitute a huge immune regulatory network. Different PRRs, such as TLR and NLR families, are indispensable to interact with DAMPs from the dying cells. After the immunological mission of neutrophils is accomplished, the apoptosis of neutrophils is very important for the withdrawal of inflammation or tissue damage. But the prosurvival of neutrophils usually aggravates the injury. Proteases, as key functional component from neutrophils, could exert a double-sided effect on the pathogenesis of respiratory diseases. They could clear the adverse factors to maintain the homoeostasis. On the other hand, their excessive secretion also contributes the injury of normal tissue. In addition, neutrophils have developed a unique NET system to play their role of double-edged sword. It can not only trap the pathogens but also amplify the inflammatory cascade. However, there are still many unanswered questions on the role of neutrophils in lung cancer, pulmonary fibrosis, cystic fibrosis, bronchitis, and bronchiolitis. Despite that the detailed pathogeneses of these diseases are fragments for now, it is believed that more and more scientific researches will connect them. Different pharmaceutical intervention had been investigated to act upon the neutrophils and manifested a promising effect to some extent. The clarification on the mechanism of traditional drugs and the development of new precise medicine, such as monoantibody and vaccine will be the following research direction. Neutrophils have the potential to be a new therapeutic target as expected in the future. Finally, understanding how neutrophils cooperate with other immune component to integrate the disease pathogenic mechanisms, and exploring how to develop novel avenues for therapeutic strategies aimed at the key pathway involved of neutrophils, will offer further insights and inform better treatment of respiratory diseases.
